# Characterisation of the Nematode Community of a Low-Activity Cold Seep in the Recently Ice-Shelf Free Larsen B Area, Eastern Antarctic Peninsula

**DOI:** 10.1371/journal.pone.0022240

**Published:** 2011-07-20

**Authors:** Freija Hauquier, Jeroen Ingels, Julian Gutt, Maarten Raes, Ann Vanreusel

**Affiliations:** 1 Marine Biology Section, Ghent University, Ghent, Belgium; 2 Alfred Wegener Institute for Polar and Marine Research, Bremerhaven, Germany; National Institute of Water & Atmospheric Research, New Zealand

## Abstract

**Background:**

Recent climate-induced ice-shelf disintegration in the Larsen A (1995) and B (2002) areas along the Eastern Antarctic Peninsula formed a unique opportunity to assess sub-ice-shelf benthic community structure and led to the discovery of unexplored habitats, including a low-activity methane seep beneath the former Larsen B ice shelf. Since both limited particle sedimentation under previously permanent ice coverage and reduced cold-seep activity are likely to influence benthic meiofauna communities, we characterised the nematode assemblage of this low-activity cold seep and compared it with other, now seasonally ice-free, Larsen A and B stations and other Antarctic shelf areas (Weddell Sea and Drake Passage), as well as cold-seep ecosystems world-wide.

**Principal Findings:**

The nematode community at the Larsen B seep site differed significantly from other Antarctic sites in terms of dominant genera, diversity and abundance. Densities in the seep samples were high (>2000 individuals per 10 cm^2^) and showed below-surface maxima at a sediment depth of 2–3 cm in three out of four replicates. All samples were dominated by one species of the family Monhysteridae, which was identified as a *Halomonhystera* species that comprised between 80 and 86% of the total community. The combination of high densities, deeper density maxima and dominance of one species is shared by many cold-seep ecosystems world-wide and suggested a possible dependence upon a chemosynthetic food source. Yet stable ^13^C isotopic signals (ranging between −21.97±0.86‰ and −24.85±1.89‰) were indicative of a phytoplankton-derived food source.

**Conclusion:**

The recent ice-shelf collapse and enhanced food input from surface phytoplankton blooms were responsible for the shift from oligotrophic pre-collapse conditions to a phytodetritus-based community with high densities and low diversity. The parthenogenetic reproduction of the highly dominant *Halomonhystera* species is rather unusual for marine nematodes and may be responsible for the successful colonisation by this single species.

## Introduction

The Larsen B ice shelf, located off the Eastern Antarctic Peninsula, began its retreat in 1995 and culminated in the break-up of 2300 km^2^ of the northern ice shelf into many small icebergs over just one week in March 2002. The total surface area of the Larsen B ice shelf decreased from 11 512 km^2^ in January 1995 to 2667 km^2^ in April 2003 [1]. The reason for this rapid disintegration was attributed to increased air temperatures, resulting in the warmest summer preceding the collapse and the occurrence of large meltwater pools on the surface of the ice, further facilitating disintegration [2], [3]. The phenomenon of ice shelf collapse is not unexpected along the Antarctic Peninsula, as this area, together with north-western North America and an area on the Siberian Plateau is amongst the fastest warming and changing regions on earth [4]. In each of these areas, mean annual temperatures increased with more than 1.5°C since 1950, compared to a global mean increase of approximately 0.6°C over the 20^th^ century [5], [6]. During the latter part of the 20^th^ century no less than seven ice shelves have retreated west off the Eastern Antarctic Peninsula [5]. Their recent disintegration as a result of global temperature rise creates unprecedented opportunities to explore the formerly inaccessible sub-ice-shelf seafloor.

Cold seeps have been observed in a variety of margin systems around the globe, at different latitudes and depths [7], [8]. Cold seep ecosystems have remained undiscovered in the Antarctic until 2005, when after the break-up of the permanent ice cover in the Larsen B area, Domack et al. [9] first discovered a possible methane seep. Characterised by an ecosystem that derives its primary metabolic energy from chemical processes instead of, or in addition to, settling phytodetrital matter from euphotic waters [10]–[12], these remarkable habitats host a unique fauna [8], [13], [14]. Cold seeps are highly patchy ecosystems due to their occurrence in a variety of geologically diverse habitats with differences in fluid origin and composition, and flow rate [7], [15]. As a consequence, specialised communities are associated with the different habitats and vertical zones found in cold-seep sediments [8]. Larger inhabitants of cold seep systems often belong to the tubeworm family Siboglinidae and bivalve families Vesicomyidae and Mytilidae, which live in symbiosis with chemoautotrophic bacteria [7], [8]. They may form extensive aggregations (so-called tubeworm fields and mussel beds) which act as a three-dimensional habitat for other invertebrates [8], [10], [16]. The presence of these characteristic seep megafauna often serves as an indication of the occurrence of high methane or sulphide levels [7]. Other habitat types found at cold seeps include microbial mats, consisting of mat-forming genera such as *Beggiatoa, Arcobacter* and *Thiothrix* [8], [10], [16]. Within cold seep systems, as well as other reducing environments, diversity tends to decrease with increasing sulphide levels which leads to the replacement of species-rich communities with low-diversity assemblies dominated by one or a few species that seem to be able to cope with the sulphidic and/or reduced conditions [12], [14], [16]–[19]. Also within the meiofauna, and more specifically the nematodes, sulphidic sediments are expected to reduce diversity and enhance dominance of a few genera or species [8], [20], whereas standing stocks may occasionally exceed background values greatly [14].

During a videographic survey in the Larsen B region in March 2005, Domack et al. [9] observed whitish material covering the seabed, assuming it consisted of sulphide-oxidising, chemoautotrophic bacteria. Such microbial mats are typical for active cold seeps where the anaerobic oxidation of methane leads to high sulphide concentrations near the sediment surface [8], [21]. An additional sign of cold-seep activity was present in the form of small mounds encrusted by bivalve shells belonging to the seep-associated vesicomyid clam *Calyptogena* sp., which lives in symbiosis with sulphur-oxidising bacteria [22].

During the austral summer of 2006–2007, the Larsen A and B areas were visited within the “Census of Antarctic Marine Life” (CAML) programme during the R/V Polarstern ANT-XXIII/8 expedition. The aim of this expedition was to obtain a synoptic overview of the area [23] with sampling at different spatial scales, for different groups of organisms and different environmental settings. Five stations were selected within the formerly permanently ice-covered Larsen A and B regions. Gutt et al. [23] investigated the benthos (mainly macro- and megafauna) in this area for the first time, while Raes et al. [3] examined the nematode communities (meiofauna). These studies provided key insights into the benthic diversity and ecology of sub-ice-shelf communities and their response to the loss of permanent ice cover. During the same expedition, samples were collected at the seep site discovered by Domack et al. [9] (hereafter referred to as Larsen B_Seep), located centrally in the Larsen B embayment at a depth of ∼820 m, in a trough formed by the Evans and Crane glaciers some 100 km from the pre-collapse (1995) ice shelf front [9], [21]. Niemann et al. [21], based on samples from the same glacier trough taken during the ANT-XXIII/8 Polarstern campaign, reported the presence of clam patches as described in Domack et al. [9] but only dead shells had been recovered this time. Furthermore, instead of the conspicuous white ‘mats’ observed by Domack et al. [9], the seabed surface had a green-greyish colour, proof of settled pelagic sediments and phytodetritus. There were no signs of gas ebullition, which implied that sulphide flux and hence seep activity had diminished over the years [21]. This was attested by the presence of only moderately elevated concentrations of thermogenic methane below 30 cm sediment depth and a slight decrease of sulphate in deeper layers (below 1 m depth). Sulphide concentrations and the rate of anaerobic oxidation of methane (AOM) increased with depth but only took place below 1 m sediment depth [21].

In this study, we characterise the meiofauna and their most abundant component, the nematodes of the Larsen B_Seep area. Usually comprising more than 90% of the meiofauna community [24], [25], nematodes present an abundant study object both in regular shelf sediments and seep environments. Two hypotheses are tested: (i) In accordance with the findings of Niemann et al. [21] the nematode assemblage and trophic signals will not point to (reduced) seep activity beneath the former ice shelf. (ii) The nematode community will be impoverished in terms of density and diversity because of long-term ice-shelf cover and concomitant lack of phytodetrital export from the overlaying euphotic surface waters compared to areas that have been exposed to phytodetrital input for a longer period of time. To investigate this, structure, diversity and trophic status of the nematode community at B_Seep was compared to those of other Larsen stations [3], other Antarctic shelf areas [24], [26] and other seeps world-wide. The current study is the first, to our knowledge, to provide data and insights on the functioning of the nematode community of the Larsen B cold seep area and contributes to knowledge on Antarctic shelf communities formerly covered by permanent ice shelves.

## Materials and Methods

### Sampling area and methods

**Figure 1:**
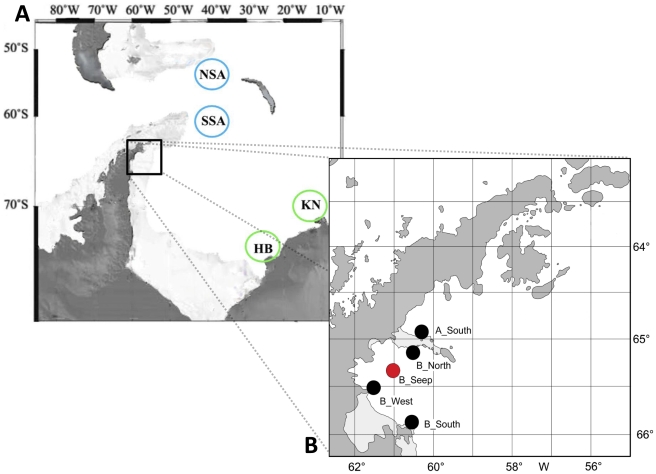
Overview of the stations. The stations sampled during the ANT-XXIII/8 Polarstern campaign are indicated on the inserted map **B**. Larsen B_Seep is indicated in red (adapted from [75]). The reference stations used for comparison are indicated on map **A**: two areas in the Drake Passage (Southern Scotia Arc (SSA) & Northern Scotia Arc (NSA) indicated in blue; [24]), two locations at the eastern Weddell Sea (Halley Bay (HB) & Kapp Norvegia (KN) indicated in green; [26]).

Sampling was conducted at the Antarctic Peninsula during the ANT-XXIII/8 Polarstern campaign in 2006–2007 in the framework of the Belgian Science Policy project BIANZO II (BIodiversity of three representative groups of the ANtarctic ZOobenthos: *coping with change*). Special attention was given to the Larsen A and B areas at the eastern side of the Peninsula, which were covered with a permanent ice shelf until 1995–2002. Larsen A lies to the north of Larsen B and both areas are separated by a narrow land/ice strip. A total of five stations was sampled for meiofauna analyses: Larsen B_South, Larsen B_Seep, Larsen B_West, Larsen B_North and Larsen A_South (Fig 1). The different stations have been free of ice cover for different periods of time (see [3]). Larsen B_South was always situated very close to the ice shelf edge, and hence subject to conditions of the open Weddell Sea, whereas the other stations have only been exposed since 2002 or later (Larsen B collapse). Larsen B_Seep is analysed in this study and was selected because of possible cold-seep activity [9], [21]. The other four stations were analysed by [3] and serve as a reference throughout this study. The Larsen B seep area lies more centrally compared to the other Larsen stations, at a depth of approximately 820 m, whereas depth ranges between 229 m and 427 m for the other stations. This difference in depth is attributed to its location within a trough formed by two glaciers [21].

Meiofauna sampling was carried out using a multicorer (MUC) device, enabling recovery of twelve cores with undisturbed sediment-water interface per deployment. The separate core tubes each had an inner diameter of 57 mm, corresponding to approximately 25.5 cm^2^ cross-sectional surface area [27]. Similar to the other Larsen stations [3], different MUC deployments (replicates) were obtained in the seep area, four of which are analysed in this study. The four analysed replicates (see Table 1) were selected based on the presence or absence of a clear sulphidic layer (3–10 cm depth in the core) with grey-black colour and typical hydrogen-sulphide smell: replicates 706/5 and 709/5 showed a grey sulphidic layer at respectively 7 and 3 cm, whereas 706/6 and 709/8 did not. Data on the different MUC deployments and analysed cores are given in Table 1 [28].

**Table 1:** Data on the different MUC deployments at Larsen B_Seep during the Polarstern ANT-XXIII/8 campaign (adapted from [28]).

Drop no.	Date	Latitude	Longitude	Depth (m)	Core no.	Analysis	Sulphidic layer	Depth of sulphidic layer
PS69/706/5	14/01/2007	65°26.09′ S	61°26.48′ W	819	3	community	Present	7 cm
					5	environmental	Present	9–10 cm
PS69/706/6	14/01/2007	65°26.10′ S	61°26.53′ W	820	5	community	Absent	
					6	environmental	Absent	
PS69/709/5	15/01/2007	65°26.09′ S	61°26.51′ W	819	4	community	Present	3 cm
					5	environmental	Present	4–5 cm
					7	stable isotopes	Present	4–5 cm
PS69/709/8	15/01/2007	65°26.07′ S	61°26.49′ W	818	3	community	Absent	
					4	environmental	Absent	

### Environmental variables

One core per deployment was analysed for grain size and photopigments (see Table 1: environmental). These cores were immediately sliced per cm, down to 5 cm sediment depth and stored at −20°C until further analysis in the lab. Grain size analysis was performed using laser diffraction (Malvern Mastersizer 2000, size range: 0.02–2000 µm) and relative percentages of the different size fractions were calculated following Wentworth [29]. The size classes used in this study include: volume weighted mean (i.e. mean grain size), clay % (4–38 µm), silt % (38–63 µm), very fine sand % (63–125 µm), fine sand % (125–250 µm) and medium sand % (250–500 µm), according to analyses carried out by [3].

Photopigment concentrations were measured with a fluorescence detector after separation using HPLC (High Performance Liquid Chromatography). Prior to measurements, pigments had been extracted from the lyophilised sediments by adding 10 ml 90% acetone. Following pigment variables are considered, in accordance with [3]: µg/g chl a (chlorophyll a), µg/g phaeopigments (i.e. the degradation products of chlorophyll a) and µg/g CPE (total Chloroplastic Pigment Equivalents), both for total sediment depth (up to 5 cm) and the upper cm separately.

### Meiofauna and nematode analysis

#### Meiofauna

One core per MUC deployment was sectioned into different sediment depth layers for meiofauna analysis: 0–1, 1–2, 2–3, 3–4, 4–5, 5–10 and 10–15 cm (see Table 1: community). These sediment slices were fixed separately in 4% buffered formalin. The meiofauna fraction (32–1000 µm, both permanent and temporal, [30]) was separated from the larger macrofauna in the lab, using a 1 mm sieve. Meiofauna was retained on a 32 µm mesh-size sieve and extracted from the sediment using density gradient centrifugation with Ludox HS-40 as a flotation medium (specific density of 1.18 g/cm^3^; [31], [32]). Samples were centrifuged (3×12 minutes at 3000 rpm) to extract all meiofauna. After centrifugation, meiofauna was stained with Rose Bengal (0.5 g/l) and stored in 4% buffered formalin. Identification at higher taxon level and counting of the metazoan meiofauna individuals in each sediment layer was carried out with a stereoscopic microscope (50x magnification) using Higgins and Thiel [33].

#### Nematodes

From each sediment layer of the four replicates, 120 nematodes were picked out randomly and transferred to anhydrous glycerol using the formalin-ethanol-glycerol technique [32]. Nematodes were then mounted on glass slides and identified to genus level with a Leica DMLS compound microscope (1000x magnification), using a pictorial key to nematode genera [34], the NeMys database [35] and other relevant literature such as [36].

Stable carbon isotope analysis was performed on Larsen B_Seep samples to test for the presence of a chemosynthetic carbon source. Additional material from the Larsen B_North station was used for comparison with a non-seep site. Sediment layers with highest nematode densities of both Larsen B_Seep (2–3 cm) and Larsen B_North (0–1 cm; [3]) were subjected to the analysis. Only specimens of the highly abundant *Halomonhystera* species in the Larsen B_Seep and Larsen B_North replicates were used, both from frozen samples (−20°C; 0–1 cm and 2–3 cm) and formalin(4%)-stored samples (2–3 cm). Sufficient nematode specimens (150–200) were picked out to obtain enough biomass for analysis and reproducible ^13^C isotope measurements (ca. 5 µg carbon per sample; [37]). Detailed information on sediment depth layer and number of nematodes picked out per sample is given in Table 2.

**Table 2:** Stable isotope analysis.

Station	Depth layer	Preservation method	# replicates	# nematodes per replicate
709/5 (Larsen B_Seep)	0–1 cm	−20°C	2	200
709/5 (Larsen B_Seep)	2–3 cm	−20°C	2	200
		Formalin 4%	3	150
718/1 (Larsen B_North)	0–1 cm	−20°C	2	200

Next to a comparison between food sources (chemosynthetic vs. phytoplankton-derived), the 2–3 cm layer of only B_Seep was used to compare between preservation methods (frozen vs. formalin-stored samples). Nematodes from frozen samples were elutriated from the sediment through density gradient centrifugation using Ludox HS-40 (see earlier; [32]) and picked out immediately to minimise decay of the carbon isotope signal [37]. They were then rinsed thoroughly with miliQ-water to remove adhering particles that might disturb the signal (e.g. detritus, sediment). All 200 specimens of each frozen sample were put in a small Aluminium (Al) cup for further analysis (2.5×6 mm, preheated at 550°C overnight to remove contaminating organic matter; [37]). The specimens from the formalin-preserved sample were rinsed twice with miliQ-water before transfer to Al-cups. Finally, the cups were dried overnight at 60°C and pinch closed.

Measurements were carried out using a CHN-analyser (Elemental Analyser – EA) coupled through an interface to an Isotopic Ratio Mass Spectrometer (IRMS, Flask 1112 series). Stable carbon isotope ratios are expressed relative to the Vienna Pee Dee Belemnite (VPDB) reference standard using the delta notation (expressed in ‰): δ^13^C = [(X_sample_ – X_standard_)/X_standard_]×10^3^, with X = ^13^C/^12^C. Each isotope value was subjected to a blank correction since obtained values are a combination of both the delta value of the organic matter in the cup (δ_OM_) and of the cup itself (δ_blank_). The blanks consisted of the same analysed Al-cups but without nematode content, and were measured at regular intervals between the actual samples. Sample signals were corrected using: δ_OM_×amplitude_OM_ = (δ_sample_×amplitude_sample_) – (δ_blank_×amplitude_blank_) in which the amplitudes and delta values of the sample and blanks are measured and the amplitude of the OM equals the amplitude of the sample minus the mean blank amplitude.

### Statistical analysis

The dataset used for further statistical analysis included nematode genera and abundance data (ind/10 cm^2^) from different locations around the Antarctic continent for comparison with the Larsen seep site (Fig 1): Halley Bay and Kapp Norvegia in the south-east Weddell Sea [26], two stations located near South Georgia and Signy Island in the Drake Passage [24] and the four other Larsen stations ([3]; new data). Due to differences in identification of the different genera of the family Monhysteridae, all genera have been grouped under this family name prior to statistical analyses, to avoid misinterpretation. The problem mainly lied within the identification of two closely-related and similar genera *Halomonhystera* and *Thalassomonhystera*.

PRIMER v6 software [38] was used to calculate Bray-Curtis dissimilarities between different stations and produce a non-metric multidimensional scaling two-dimensional plot (nMDS) and a one-way crossed Analysis of Similarities (ANOSIM test). A SIMPER (Similarities of Percentages) analysis was performed to determine which genera contributed most to the observed differences. All nematode abundance data were standardised (to account for differences in sample sizes) and square-root transformed (to down-weigh the importance of very abundant genera without losing the influence of rarer genera) prior to analysis. PRIMER v6 was also used to calculate various diversity indices to compare generic diversity between different stations. Indices included in this study are: Hill's indices N_0_, N_1_ and N_∞_ [39], Pielou's evenness index J', Shannon-Wiener diversity index H'_loge_ and the expected number of genera EG(51) for a sample size of 51 individuals.

## Results

### Characterisation of the site

**Figure 2:**
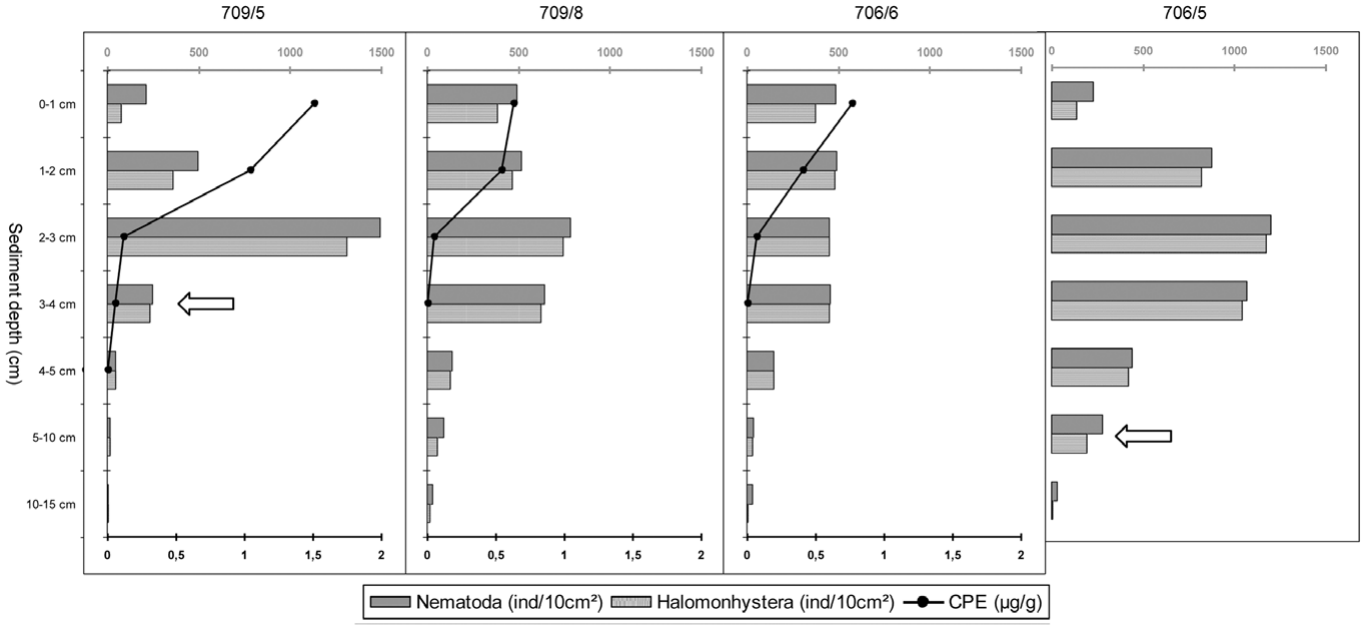
Vertical profiles of nematode densities, *Halomonhystera* densities and CPE concentration per sediment layer. Scale bars have been made uniform for comparison. White arrows indicate the position of the sulphidic layer as observed upon sampling [28].

At Larsen B_Seep, some sediment cores exhibited a clear sulphidic layer between 3 and 10 cm sediment depth (see Table 1; Fig 2), characterised by a typical sulphide smell and dark blue-grey colour ([28]; personal observations). Empty shells of the cold-seep clam *Calyptogena*, also observed by [9], were still present at the time of sampling [21], [28].

An overview of sediment characteristics is provided in Table 3. All Larsen B_Seep replicates were characterised by similar muddy clay-silt sediment. Mean particle size ranged from 9.62 µm at replicate 709/8 to 11.03 µm at replicates 706/5 and 709/5, yielding an overall mean grain size of 10.36±0.78 µm (volume weighted mean). Silt was dominant with an average contribution of 65%. Together with the clay fraction (34% average), pellite (silt + clay) was the dominant size class. Only minor fractions of sand were present (<1%). The same trends were observed for the upper cm separately (Table 3).

**Table 3:** Overview of sediment characteristics for the different Larsen B_Seep replicates and averaged for the seep station.

Larsen B_Seep	Sediment layer	chl a (µg/g)	phaeo (µg/g)	CPE (µg/g)	Volume weighted mean (µm)	clay%	silt%	very fine sand%	fine sand%	medium sand%
**709/5**	all layers	0.378	0.166	0.544	11.03	31.15	68.19	0.50	0.12	0.04
	upper cm	0.934	0.570	1.504	13.90	26.67	71.63	1.09	0.40	0.22
**709/8**	all layers	0.180	0.063	0.243	9.62	36.44	63.16	0.40	0.00	0.00
	upper cm	0.452	0.179	0.631	11.03	32.40	66.83	0.76	0.01	0.00
**706/5**	all layers	−	−	−	11.03	34.01	65.04	0.79	0.10	0.06
	upper cm	−	−	−	17.02	25.43	71.45	2.38	0.45	0.29
**706/6**	all layers	0.149	0.098	0.247	9.75	35.71	63.80	0.38	0.08	0.03
	upper cm	0.400	0.360	0.760	12.79	30.08	68.53	0.99	0.27	0.13
**Average**	all layers	0.24±0.12	0.11±0.05	0.35±0.17	10.36±0.78	34.33	65.05	0.52	0.08	0.03
	upper cm	0.60±0.29	0.37±0.20	0.97±0.47	13.69±2.52	28.65	69.61	1.31	0.28	0.16

Note that data on pigment concentrations are missing for replicate 706/5.

Chloroplastic Pigment Equivalents (CPE) ranged from 0.243 µg/g for replicate 709/8 to 0.544 µg/g for 709/5 (mean values over 5 cm sediment; Table 3) with higher concentrations in the core where a sulphidic layer had been observed (709/5). Average CPE was 0.345±0.172 µg/g of which 0.236±0.124 µg/g was chlorophyll a. Pigment concentrations decreased rapidly throughout the sediment column, with total CPE ranging between 0.6 and 1.5 µg/g in the upper cm and decreasing to zero after the third (706/6 and 709/8) or fourth (709/5) cm (see Fig 2). Vertical CPE profile for replicate 706/5 is missing since values for the different sediment depth layers were not available. Contribution of phaeopigments was comparable for the different replicates, ranging between 25.9 and 39.8% of total CPE concentration.

### The benthos at Larsen B_Seep

Table 4 gives an overview of the meiofauna composition and densities for the four Larsen B_Seep replicates. A total of 8 metazoan meiobenthic taxa was recovered at Larsen B_Seep, of which nematodes were the dominant taxon (>90%), followed by harpacticoid copepods and their nauplii larvae (Table 4). Other taxa contributed less than 1% to total abundance and included Polychaeta, Ostracoda, Isopoda, Kinorhyncha, Halacarida, and Rotifera. Highest meiobenthic densities were observed at replicate 706/5 (4205 individuals per 10 cm^2^) and lowest at 706/6 (2220 ind/10 cm^2^), with an average density of 2988±851.5 ind/10 cm^2^ over the four replicates (see Table 4). Nematode densities followed the same pattern with the highest value at replicate 706/5, intermediate values at replicates 709/5 and 709/8 and the lowest value at replicate 706/6, leading to an average nematode density of 2863±877 ind/10 cm^2^. Most meiobenthic taxa resided in the upper cm where CPE concentrations were highest (see Fig 2), except for the nematodes, which showed a deeper density maximum at 2–3 cm depth in three out of four replicates (not the case for replicate 706/6 but also there, densities were high until after 3 cm depth; Fig 2). A total of 66 different nematode genera were identified over the four replicates. Total number of genera (N_0_) was highest at replicate 706/6 (32) and lowest at 709/5 (22). Yet, the expected number of genera for 51 individuals showed the opposite trend with highest numbers for 709/5. Other diversity indices (Shannon-Wiener H'loge and Hill's N1) as well as evenness (N_∞_, J') were highest for replicate 706/5. Genera with relative abundance >1% are given in Table 5. All replicates were dominated by a single species of *Halomonhystera* (relative abundance 79.57–86.03%) of which only juveniles and females were recovered and which was dominant in all sediment depth layers of the B_Seep replicates (Fig 2) except for the deepest five cm's (10–15 cm) of replicates 706/5 and 706/6 where an undescribed genus of the family Ethmolaimidae and the genus *Theristus* were most abundant, respectively. Few other genera surpassed the 1% limit as they consisted of only one or a few specimens.

**Table 4:** Meiofauna composition (in percentages) and densities (in individuals per 10 cm^2^) of the four Larsen B_Seep replicates.

Replicate	Nematoda (%)	Copepoda (%) (Harpacticoida)	Nauplii (%)	Other (%)	Meiofauna density (ind/10 cm^2^)	Nematode density (ind/10 cm^2^)
709/5	92.76	2.77	3.56	0.91	2718	2522
709/8	96.08	1.55	1.49	0.88	2809	2699
706/5	98.11	0.90	0.57	0.42	4205	4125
706/6	94.88	1.89	2.29	0.94	2220	2107

**Table 5:** Relative abundance of dominant genera at the different Larsen B_Seep replicates (relative abundance >1%).

PS69/706/5		PS69/706/6		PS69/709/5		PS69/709/8	
Genus	%	Genus	%	Genus	%	Genus	%
*Halomonhystera*	79.57	*Halomonhystera*	83.73	*Halomonhystera*	82.39	*Halomonhystera*	86.03
*Ethmolaimidae gen. nov.*	9.44	*Theristus*	5.21	*Daptonema*	4.70	*Monhystrella*	2.22
*Monhystrella*	1.55			*Monhystrella*	1.57	*Daptonema*	1.75
*Daptonema*	1.08					*Stephanolaimus*	1.75

To test for food preference of the dominant *Halomonhystera* species, a stable carbon isotope analysis was performed which yielded the δ^13^C values listed in Table 6. Values are averaged over the different replicates for the samples and are corrected for a blank with average delta value of −25.385‰ and average amplitude of 54.53. Average ^13^C delta for the formalin-stored samples (709/5, 2–3 cm) was −21.97±0.86‰. The ^13^C isotope signal was slightly more depleted for the frozen samples (−23.27±0.31‰). Average δ^13^C for the B_North replicate (0–1 cm) was −22.52±0.36‰ and slightly lower for the B_Seep replicate (−24.85±1.89‰), but note that the standard deviation for the latter replicate is much higher.

**Table 6:** Calculated delta values (in ‰, ± standard deviation) for the C-13 stable isotopes.

Replicate	Station	Average delta*
709/5/2–3 cm formalin	B_Seep	−21.97±0.86
709/5/0–1 cm frozen	B_Seep	−24.85±1.89
718/1/0–1 cm frozen	B_North	−22.52±0.36
709/5/2–3 cm frozen	B_Seep	−23.27±0.31

*Delta values are based on two replicate measurements each and are blank corrected.

## Discussion

Larsen B_Seep is a remarkable area for two reasons. Firstly, it has a long history of ice-shelf coverage prior to the rapid disintegration of Larsen B in 2002. And secondly, cold-seep activity has been assumed in the past [9], but has strongly declined over time [21]. Both phenomena are very likely to influence the small motile benthic organisms. The meiofauna and nematode community structure are therefore compared with other areas world-wide and the Antarctic shelf in particular. Subsequently, we will focus in this discussion on the impact of reduced cold-seep activity and permanent ice-cover loss.

### The benthos at Larsen B_Seep: Business as usual?

**Figure 3:**
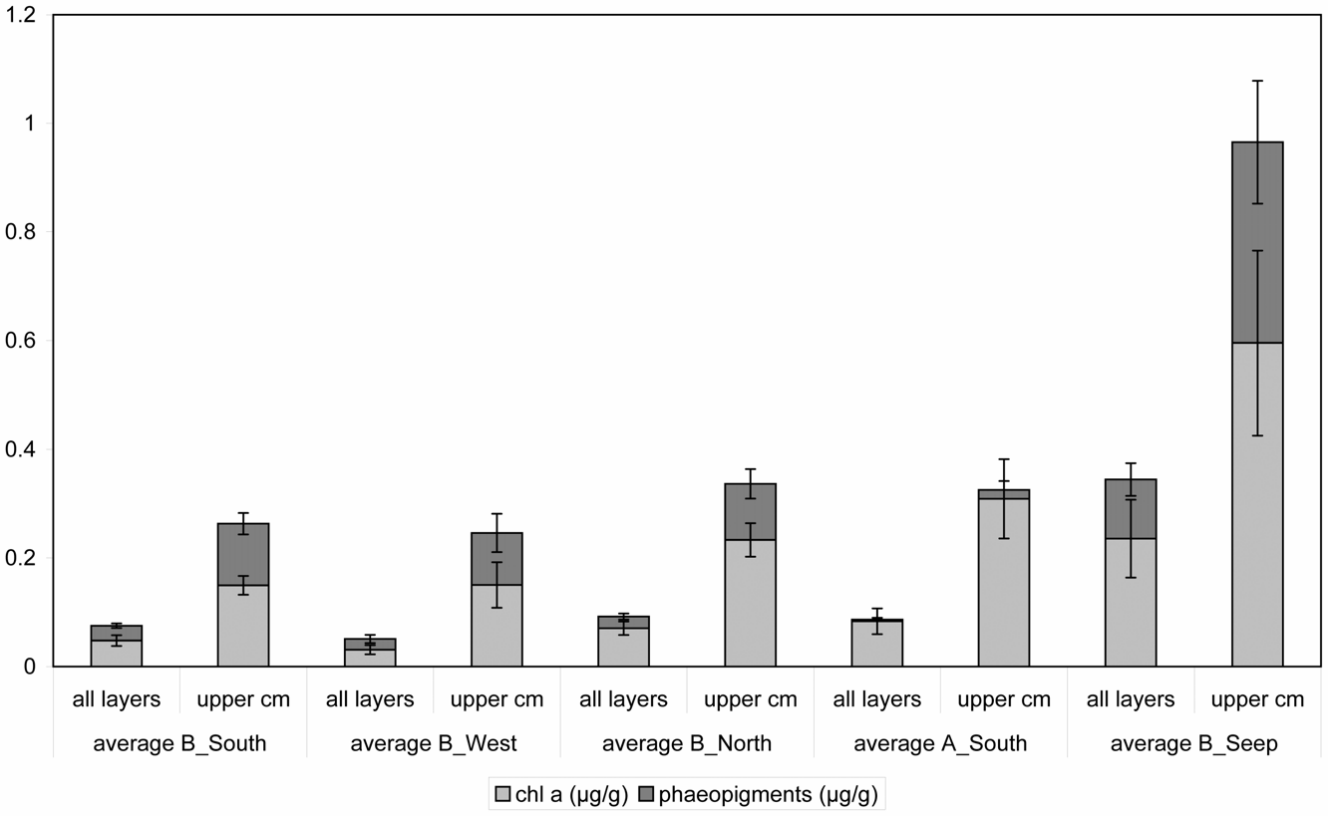
Pigment concentrations of the five Larsen A and B stations. Concentrations are given both for the entire sediment column and the upper cm separately (in µg/g). Bars represent standard errors.

In general, meiofaunal standing stock and distribution in the sediment is to a large extent determined by the amount of utilisable organic matter in the sediments [40]–[42], which in turn is controlled by sedimentation rate, degradation in the water column, and other benthic processes. Higher CPE concentrations in the sediment, especially chlorophyll a (as a measure of freshness of the organic matter), are indicative of a higher primary production in the euphotic zone, in combination with a shallower water depth. Because pigment concentrations and hence food availability tend to decrease with increasing water depth and distance to the coast [26], [42]–[44], meiofaunal densities often decline when moving from the shallows towards the deeper abyss [31], [40], [42]–[45]. At Larsen B_Seep, total phytopigment concentrations were the highest for the whole Larsen region studied so far (Fig 3), which was also observed by [23] during the same ANT-XXIII/8 campaign (data provided by Isla & Sañé Schepisi). Enhanced CPE concentrations at B_Seep relative to adjacent, even shallow, stations can partly be explained by its location within a trough formed by two glaciers. This trough may act as a trap accumulating food deposition from the surface waters [23], [44].

However, when comparing sediment CPE levels for the entire Larsen area with other Antarctic shelf areas, it became clear that pigment concentrations were generally quite low in the Larsen A and B embayments [23], [46]. Concentrations at B_Seep (0.24–0.54 µg/g) were within the same order of magnitude as in the Ross Sea at a depth of ∼400–550 m (0.25±0.24 µg/g−0.45±0.37 µg/g; [42]), which was according to these authors up to 5 times lower than in equally deep sediments in the Weddell Sea (Kapp Norvegia and Halley Bay; [40]). Also compared to other regions world-wide at similar water depths, CPE concentrations were rather low ([47]: 1.45–4.48 µg/g at depths between 533 and 2401 m in the Ionian and Aegean Seas; [48]: 0.467–1.585 µg/g at a depth of 750 m in the South Sandwich Trench).

**Figure 4:**
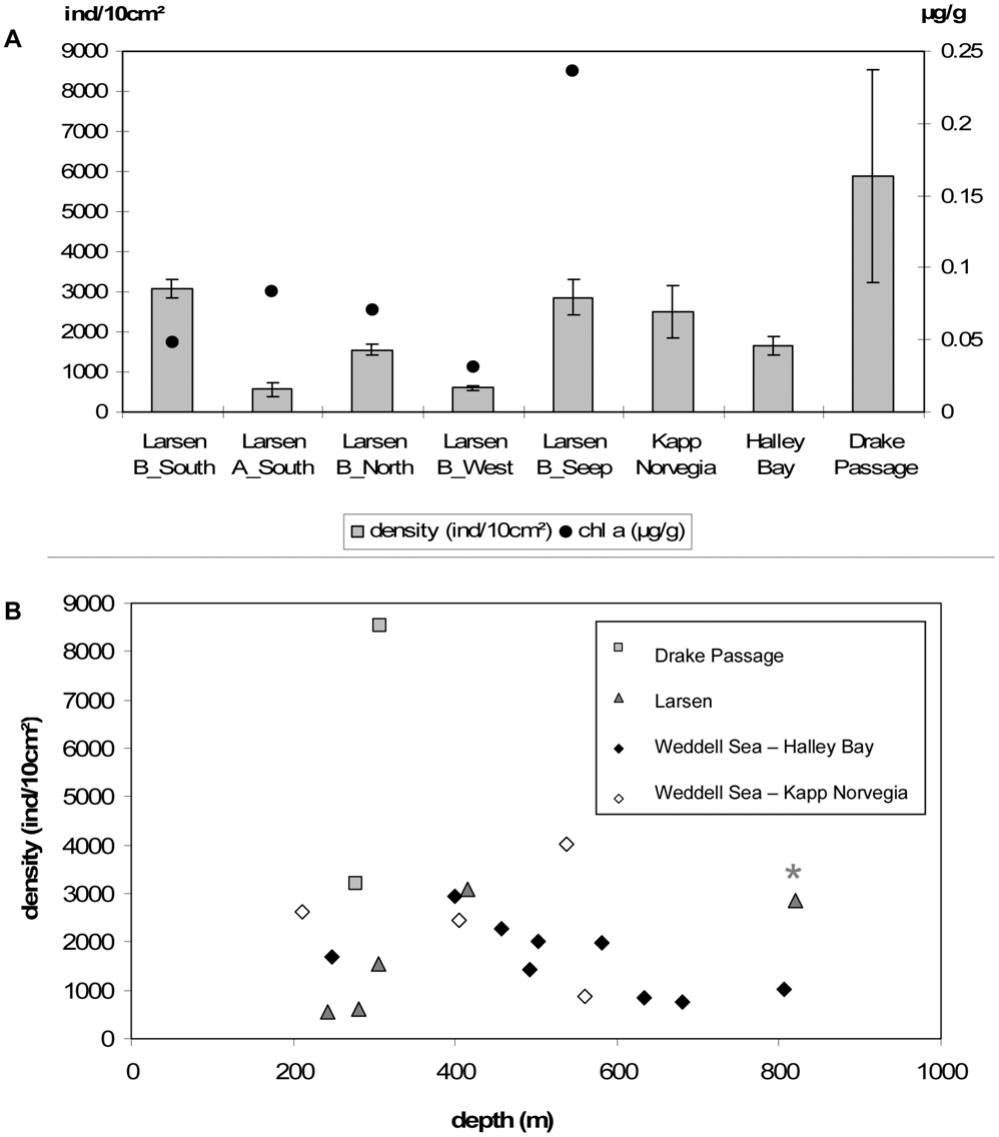
**A.** Nematode densities in comparison with other Larsen and Weddell Sea stations. Average nematode densities (in ind/10 cm^2^, bars) and chlorophyll a concentrations (µg/g, dots) for the different Larsen stations and reference areas. Bars represent standard errors. Note that for the reference stations, pigment data are not shown because they were expressed in µg/cm^3^ and hence could not be compared. **B.** Nematode densities (ind/10 cm^2^) for the different stations relative to their depth (0–1000 m). The position of the Larsen B_Seep station is marked with an asterisk*.

Although CPE values were low in the Larsen region, average meiofauna density at Larsen B_Seep was within the higher range found at other seasonally ice-free shelf locations in the Weddell, Scotia and Ross Seas [40], [43], [48] (see Fig 4A & B). At B_Seep, densities varied substantially between the different replicates (Table 4) but such microscale variation could be ascribed to small-scale differences in microtopography and physical structure of the habitat, as well as patchiness of food availability in the sediments [40], [42], [49]. Since nematode densities at B_Seep were higher compared to shallower Larsen stations and reference stations in the Weddell Sea (Fig 4B), they do not seem to conform to the general pattern of decrease in abundance with water depth. Highest nematode densities and CPE concentrations at B_Seep within the Larsen area could indicate that higher nematode densities are a consequence of this elevated food input. However, when put in a broader geographical context, higher densities at B_Seep relative to Weddell Sea regions of similar depth cannot simply be explained by assuming higher food input in the Larsen B_Seep area.

Not only do organic matter concentrations generally decrease with water depth, also within the sediment they tend to decline with depth from the surface to subsurface layers (due to microbial degradation [3], [44], [48]), and this is often reflected in meiobenthic densities [24], [48]. Within the Larsen B_Seep replicates, CPE concentrations were indeed highest in the upper cm of the sediment column and rapidly decreased to zero after ∼3–4 cm depth (Fig 2). Abundance of most meiofauna taxa of Larsen B_Seep followed this pattern, with highest densities in the topmost sediment layer. Meiofauna and nematode abundance were also greatest in the surface layers of other Larsen and Antarctic shelf stations, implying a dependence upon fallout of phytodetritus as dominant food source. The nematode community at Larsen B_Seep however, showed a substantially different pattern, with density maxima at 2–3 cm sediment depth (not for replicate 706/6), most prominent in both cores where a sulphidic layer was noticed (Fig 2). Occurrence of nematodes at greater sediment depth is often related to deeper penetration of food, e.g. next to burrowing structures created by macro- and megafauna, where chlorophyll and phaeopigments can infiltrate into deeper sediment layers [50], [51]. Burrows of infauna might also impact the oxygen penetration into the sediment, hence allowing deeper occurrence of nematodes [52]–[54]. However, CPE concentrations showed no sign of deeper penetration into the sediment (Fig 2), precluding availability of fresh food sources to the infauna in deeper layers. It is therefore unlikely that bioturbation and consequent redistribution of phytodetrital matter were ultimately responsible for the deeper nematode standing stocks. Macrobenthic densities at B_Seep were low but megabenthic densities were relatively high compared to the poorest station (in terms of density and diversity) B_West due to the occurrence of an abundant deep-sea holothurian *Elpidia glacialis* [23]. The same species was present in dense herds in the Molloy Deep site [55] where it was assumed to be responsible for a high predation pressure on the metazoan meiofauna, resulting in (weak) subsurface maxima. Finally, enhanced bottom currents could also invoke a downward movement of meiofauna because of constant disturbance of the upper sediment layers [56]. However, only nematodes seemed to prefer the deeper sediment layers, so that sediment disturbance by increased bottom currents probably has no real impact on the metazoan meiofauna at Larsen B_Seep.

Both in terms of total densities as well as vertical distribution in the sediments, the nematode community at B_Seep did not reflect generally observed patterns of decreasing density with water depth and sediment depth. Also general metazoan meiofauna taxon richness was rather low (8 taxa) compared to similar depths in other Antarctic regions (see [42] and references therein; [43] found a mean of ∼11 taxa per station, 16 taxa in the richest station). In all replicates, nematodes were by far the most dominant taxon, which is consistent with results from other studies in Southern-Ocean shelf areas and at bathyal depths [3], [26], [40]–[44], [48], [57]. The other observed higher taxa were typical inhabitants of soft bathyal and abyssal sediments and have been recovered on several occasions from Antarctic sediments [44]. Similar patterns in meiofauna composition have been described in cold-seep habitats as well [12], [17].

**Figure 5:**
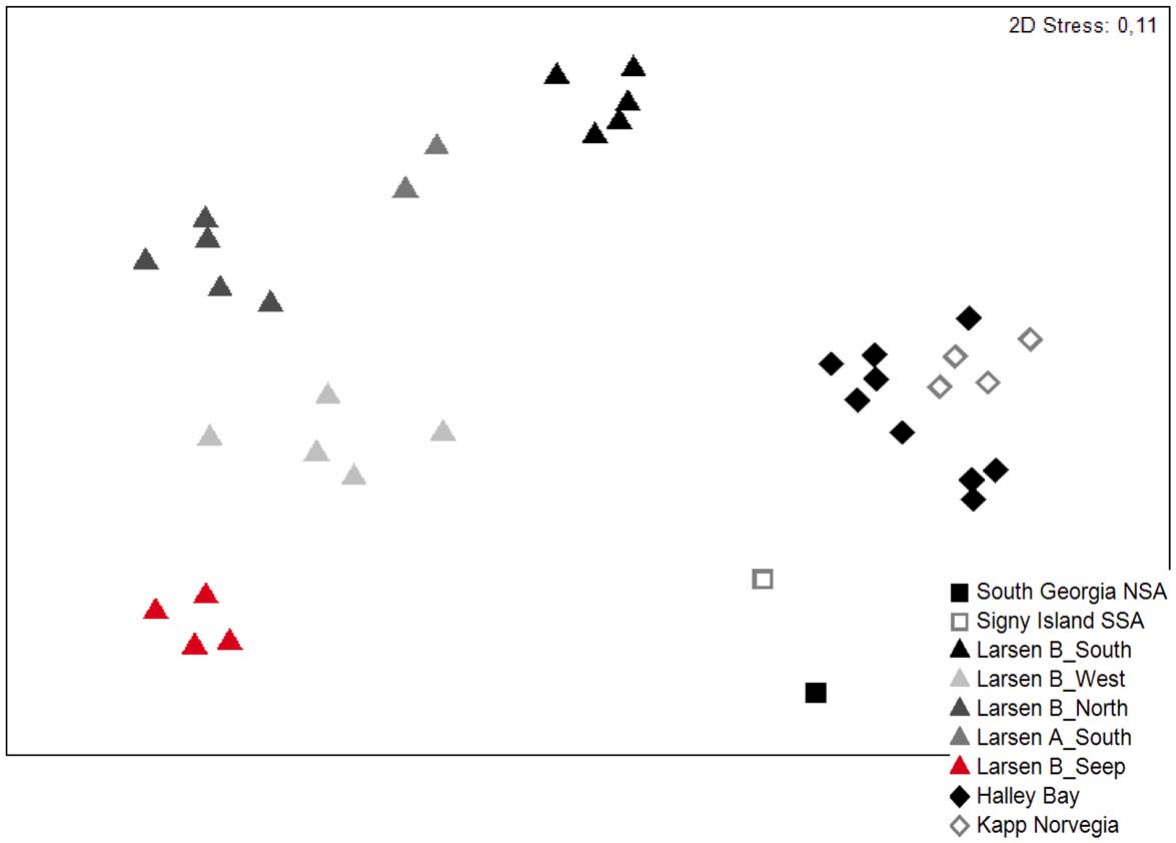
nMDS plot. The plot is based on Bray-Curtis similarities of the Larsen replicates ([3]; this study) and the reference data of the Weddell Sea [26] and Drake Passage [24]. Data have been standardised and square-root transformed prior to analysis. The stress value in the upper right corner indicates the goodness-of-fit of the MDS plot.

Meiofauna in general and the nematode community at B_Seep were depauperated compared to other Antarctic areas (see Table 7 for an overview of the average diversity values for the different areas). Low diversity at B_Seep in terms of H'loge and EG was attributed the absolute dominance of one *Halomonhystera* species (80–86%), family Monhysteridae. The same species was also found in the other Larsen stations, although with lower relative abundances (9–58%; [3]). Figure 5 shows the nMDS plot based on genera composition data, including all Larsen stations as well as other shelf stations from the Weddell Sea and Drake Passage known from literature. The high contribution of Monhysteridae to total nematode densities at B_Seep was the main factor (as revealed by SIMPER analysis) explaining on one hand the higher similarity with B_West and B_North (41.11% and 43.45%, respectively), and on the other hand the larger differences in community composition with B_South (dissimilarity of 78,22%), A_South (dissimilarity of 68.91%) and other Weddell Sea regions (dissimilarity of 75.09% with Drake Passage average and 79.42% Weddell Sea average) [24], [26], where the genus *Halomonhystera* and the family Monhysteridae are rather sparse. Overall, nematode community structure was significantly different between different shelf regions, based on the one-way ANOSIM analyses (overall R = 0.97 and *p* = 0.001 for 7 groups (5 Larsen stations + Weddell Sea + DP); R between 0.904 and 1 for all pairwise comparisons; higher R values indicate higher dissimilarity between sites).

**Table 7:** Diversity indices (average ± standard deviation) of the different Larsen stations compared to the Kapp Norvegia, Halley Bay (Weddell Sea) and Drake Passage average.

	B_South	A_South	B_North	B_West	B_Seep	Kapp Norvegia	Halley Bay	Drake Passage
**N_0_**	27.80±1.48	28.00±7.07	12.40±2.30	20.00±4.53	25.75±4.35	62.75±5.62	52.33±11.50	43.5±2.12
**J'**	0.75±0.02	0.72±0.05	0.54±0.05	0.49±0.05	0.24±0.03	0.81±0.03	0.85±0.03	0.76±0.003
**EG(51)**	16.44±2.32	18.29±2.99	8.26±1.69	12.13±3.06	4.48±0.59	23.59±2.97	25.69±4.23	17.58±0.74
**H'_loge_**	2.48±0.07	2.40±0.35	1.34±0.15	1.48±0.26	0.76±0.08	3.34±0.17	3.34±0.27	2.86±0.05
**N_1_**	11.99±0.83	11.33±3.86	3.85±0.58	4.50±1.18	2.15±0.17	28.65±4.81	29.29±8.31	17.41±0.86
**N_inf_**	3.14±0.37	3.54±1.20	1.76±0.15	1.75±0.21	1.17±0.04	7.07±2.27	8.05±1.40	4.21±0.31

In summary, the results show that there are significant differences between the nematode community of the Larsen B_Seep station and other non-seep Larsen stations [3] and other reference areas in the Weddell Sea [26] and Drake Passage [24]. Since local environmental factors can only partly explain the observed differences, these results were examined in the light of both reduced cold-seep activity and recent ice-shelf collapse.

### Reduced cold-seep activity

Our first hypothesis is that we assume to find no indication of (reduced) seepage at the nematode level. At first sight, there were no signs of typical seep-associated megafauna, except for dead, empty *Calyptogena* shells. The presence of these *Calyptogena* shells was one of the main reasons to revisit the exact sampling location of Domack et al. [9], and (although dead) an indication of higher seep activity in the past [21]. Yet, the observation of dark grey-coloured sulphidic layers in some of the replicate cores at B_Seep suggested that a certain level of cold seep activity was still present. Cold seeps, as well as other reducing deep-sea environments such as hydrothermal vents, contrast with generally observed trends in meiofauna and nematode structure of phytodetritus-based benthic environments, as did the nematode community at B_Seep.

Previous studies in a variety of cold-seep ecosystems world-wide have shown that nematodes often represent the dominant group in meiobenthos [17], [18], [58] and that community composition in sediments with high sulphide levels is mostly dominated by one or a few successful species that are able to thrive in the reduced but organically-enriched sediments. This leads to high densities (sometimes accompanied by subsurface distribution maxima) but diminished species richness [12], [17], [19], [56], [58], [59]. In bacterial mats consisting of the sulphur-oxidising genus *Beggiatoa*, recovered from the Håkon Mosby Mud Volcano in the Barents Sea (HMMV, ∼1280 m water depth), Van Gaever et al. [12], [17], [18] reported an extremely dense (>11,000 ind/10 cm^2^), mono-specific nematode community dominated by *Halomonhystera disjuncta.* In general, Monhysteridae are deep-sea generalists that are known to dominate extreme environments such as hydrothermal vents [20], [60]–[62] and seeps [16], [17] and *Halomonhystera* is a cosmopolitan genus that has been recovered from various marine sediments [31], including sulphidic sediments in shallow [31] and deeper [18], [62] areas. All of the above-mentioned characteristics of seep meiobenthos are found in the nematode community at Larsen B_Seep, with densities which were greater compared to the other Larsen stations, but not as high as for the HMMV. Although the dominant seep nematodes are not endemic for these habitats, they may show some traits that apparently help them to survive in these harsh environments: parental caring in *H. disjuncta* [17], symbiotic bacteria and absence of mouth and gut in *Astomonema* [19]. In this aspect, the presence of only juveniles and females of the dominant *Halomonhystera* species in the B_Seep samples was striking. Nematodes are known to be able to express different reproductive strategies which can be triggered by their specific environment (e.g. ovovivipary: [17] and references therein). Furthermore, many terrestrial and plant-parasitic nematodes are able to reproduce autotokously (i.e. production of progeny by a single parent, including hermaphroditism and parthenogenesis; [63]). Such methods of asexual reproduction are not regularly observed in marine free-living nematodes [63]. Within freshwater free-living nematodes, a number of studies [64]–[66] have shown that the contribution of males to the community age structure is often minimal (between 0–15%). The main part of the population often consists of juveniles (44–64%) and females (19–48%) [64], [65]. In the study by Beier and Traunspurger [64] no males were found for the dominant species *Eumonhystera filiformis/vulgaris*, which is also a member of the Monhysteridae. The authors suggested that most nematodes in the studies stream were reproducing parthenogenetically, leading to fast population growth, thus allowing the nematodes to be successful in this lotic system. The success of a single *Halomonhystera* species at B_Seep as reflected by its dominance and high densities may have followed out of a parthenogenetic reproduction strategy, although more study is required to investigate this phenomenon. Nevertheless, fast population growth may explain the unusual high numbers and high dominance of this single species in this special environment.

High densities of the *Halomonhystera* species furthermore suggest that it may be able to make use of chemosynthetically-derived food. If the *Halomonhystera*-dominated community at B_Seep depended upon a chemosynthetic food source, either by direct grazing on free-living chemoautotrophic bacteria or indirectly via symbiotic interactions, stable isotope signals of *Halomonhystera* specimens from B_Seep and B_North (as a control) should reveal the origin of their main source of food. Based on the ‘you are what you eat’-principle [67], nematode communities that depend on a chemosynthetic food source should exhibit much greater ^13^C-depletions compared to benthos that is entirely dependent upon heterotrophic consumption of phytoplankton-derived organic carbon [37], [68]. A number of isotope studies in cold seep ecosystems already revealed a link between nematodes and a chemosynthetic carbon source. For instance, Van Gaever et al. [18] showed that *Halomonhystera disjuncta* in the bacterial mats at HMMV was feeding on the free-living chemoautotrophic bacteria present.

For Larsen B_Seep and B_North, all ^13^C isotope values lay between −25 and −22‰, for both replicates, for the different sediment layers and for the different preservation methods (Table 6). These values are comparable to those for phytoplankton-derived organic matter (ranging −25 to −16‰ approximately; [19], [69]; calculated mean of −21.1‰ (SE 2.4) by Currin et al. [67] based on results of different studies) and suggest that nematodes feed on phytodetritus settling from the water column. Apparently, differences in preservation method (formalin vs. frozen samples) did not have any significant effect on isotopic values as already shown by earlier studies [69]–[72].

Coming back on our first hypothesis, i.e. there are no indications of (reduced) cold-seep activity in the Larsen B area, the results leave scope for debate. On one hand, stable carbon isotope values indeed point out that the nematodes did not depend on chemosynthesis for their carbon source at the time of sampling. On the other hand, nematode community traits, such as high densities, prominent dominance of one species and deeper standing stocks, resemble the situation at other cold seep ecosystems world-wide. Therefore, effects of reduced cold seepage on the meiofauna level cannot be ruled out.

### The benthos after ice-shelf collapse

The second hypothesis stated that the nematode community at B_Seep would be impoverished as a result of the long coverage with a thick ice shelf. The recent loss of ice cover in the area has had a profound effect on the food regime with a shift from oligotrophic, pre-collapse conditions to the development of summer phytoplankton blooms in the surface waters above the previously ice-covered sediments [23]. In general, the Southern Ocean is characterised by very short, but intense summer phytoplankton blooms which depend on the melting of the pack ice in spring and, thus, can exhibit considerable regional variation [26], [40], [69]. Blooming period is shortest and least predictable in the high latitude environments such as the Weddell Sea, including the Larsen areas. Benthic communities in Antarctic waters are almost entirely dependent upon this summer food supply, either by direct vertical transport of phytodetritus from the surface or indirectly by lateral transport through near-bottom currents that bring food particles from other seabed regions to the site [73]. It seems that especially nematodes are very sensitive to changes in food availability [42], [44]. Therefore, the loss of ice cover and subsequent enhancement of direct vertical flux of food particles to the seabed may have had a positive effect on meiofauna and nematode abundance in the Larsen area. During previous conditions of long ice-shelf coverage (>10,000 years), the only source of photosynthetically derived food was probably related to advective transport by bottom currents from other areas in the open Weddell Sea [3], [73]. Pre-collapse oligotrophic conditions in the inner Larsen embayment were reflected in the nematode community of the inner station B_West, where Raes et al. [3] found low nematode densities, low genus richness, and one highly dominant genus (*Halomonhystera*), all typical features of recent colonisation. Since B_West became ice-free after 2002 and colonisation of newly opened benthic patches might take several decades (mainly because nematodes exhibit passive dispersal by bottom currents [3], [74]), the nematode community was still in an early stage of recovery or the (re)colonisation process and therefore depauperated. The same observation was made for the other inner stations, B_North and A_South, although density and diversity differences did occur (probably due to local environmental conditions [3]). The above-mentioned ice-collapse related features (timing of ice cover loss, food availability and colonisation rate) of the different Larsen stations were the reason for the seemingly contradictory observations that densities within the Larsen area increased with water depth (see earlier; Fig 4B), as opposed to general expectations. But how do these observations relate to the Larsen B_Seep station?

Instinctively, if we look at its central position within the Larsen B embayment (Fig 1) and its timing of ice cover loss (2002) without taking into account the presumed cold seep activity, one would expect to find that the nematode community at B_Seep is most similar to the other impoverished inner stations B_West and B_North. Indeed, community structure was most comparable to those two stations in terms of the dominant *Halomonhystera* species and low diversity (Table 7). Hence, similar patterns of low diversity and *Halomonhystera* dominance at B_Seep may be indicative of the relatively recent loss of ice cover there and the presence of an early stage of colonisation. However, nematode densities were much higher than in B_West and B_North and were most similar to those recorded at B_South. The arguments used to explain increased densities at B_South (i.e. location near ice-shelf edge and open Weddell Sea [3]) cannot be used in the case of B_Seep because of its larger distance to the open Weddell Sea. Also subsurface maxima in nematode abundance cannot be explained by ice-shelf disintegration.

It seems that the long coverage with the Larsen B ice shelf indeed resulted in a poor nematode community at B_Seep in terms of diversity and dominance. Yet questions remain regarding high observed densities and extraordinary vertical profiles.

### Conclusion

Throughout this study we came across some seemingly contradicting results regarding the nematode community of Larsen B_Seep. Both recent ice-shelf disintegration and past cold-seep activity bring plausible explanations for the observed patterns in nematode diversity, density and composition. The results show that the current, impoverished nematode community may have resulted from both the long-term ice coverage in the area, as well as the proliferation of one opportunistic species to cold seep conditions in the past (although we cannot be certain about this latter aspect). The high density - low diversity nematode community is most likely the result of large-scale ice shelf collapse and subsequent increased food input from the surface waters. Stable carbon isotope values indicated that the dominant *Halomonhystera* species at B_Seep is indeed depending on phytodetritus as a food source. This species seems to be very well adapted to rapid colonisation, making use of parthenogenetic reproduction, and therefore dominates the entire community. At the same time, there are indications that cold-seep activity may have shaped the community at B_Seep and ice-shelf removal has helped the ecosystem to shift towards a phytodetritus-based community.

Today, many uncertainties remain regarding the structure and ecology of benthic communities at the Larsen B_Seep site. This study aimed at revealing some of the patterns and processes associated with this unique area by providing a first assessment of the meiobenthos. Ice shelf disintegration has made the study of these poor-known communities feasible and future effort in the area, at all trophic levels, will be needed if we are to fully understand the functioning of this remarkable ecosystem.
